# Theranostic Probes for Cancer Imaging

**DOI:** 10.1155/2017/1863803

**Published:** 2017-10-18

**Authors:** Fu Wang, Lei Zhu, Kai Yang, Dongkyoo Park

**Affiliations:** ^1^School of Life Science and Technology, Xidian University, Xi'an 710071, China; ^2^Department of Surgery, Emory University School of Medicine, Atlanta, GA 30322, USA; ^3^School of Radiation Medicine and Protection & School for Radiological and Interdisciplinary Sciences (RAD-X), Soochow University, Suzhou 215123, China; ^4^Department of Radiation Oncology, Emory University School of Medicine and Winship Cancer Institute of Emory University, Atlanta, GA 30322, USA

This special issue is dedicated to theranostic studies including original research articles and review articles, which integrate specialized diagnosis and therapy using the various theranostic probes with an emphasis on cancer research. The new term “theranostics” was coined by combining two words: therapeutic and diagnostic. More specifically, therapeutic strategies including photodynamics, chemotherapy, gene therapy, hyperthermia, and radiation are integrated with one or more diagnostic imaging techniques such as computed tomography (CT), magnetic resonance imaging (MRI), position emission tomography (PET), single photon emission computed tomography (SPECT), near-infrared (NIR) fluorescence imaging, and ultrasonography to develop molecular diagnostic tests and targeted therapeutics in clinics, resulting in the advance of personalized medicine. Therefore, these cutting-edge technologies allow us to simultaneously diagnose, image, and treat cancer and monitor the response of cancer treatment.

In this special issue, we invited colleagues worldwide who have been exploring cancer research for years, to discuss and report cutting-edge research in the use of nanomaterials for theranostics applications.

In one of the articles, Y.-Y. Ma et al. evaluate the potential of theranostic nanoparticles as a nanoplatform to load targeted molecules for both imaging and therapeutics. They also discuss the contributions of modern nanoparticles to accurate tumor image and effective tumor treatment.

In another article, W. Wang et al. successfully show that ^64^Cu-labeled anti-human AXL antibody (^64^Cu-anti-hAXL) is a useful probe for microPET/CT imaging in Triple-Negative Breast Cancer (TNBC). AXL downregulation by inhibiting heat shock protein 90 (HSP90) in TNBC* in vivo* was clearly visualized by microPET/CT.

N. H. Alamdari et al. analyze a bio-hybrid material for early diagnosis and treatment of cancer using MRI. Gd(III)-Anionic Linear Globular Dendrimer-Asparagine designed and developed by Alamdari et al. showed the promising anticancer activity with no significant side effects* in vitro* and* in vivo*.

The article by M. Chen et al. developed a CD44-targeted polymeric hyaluronic acid nanoparticle (HANP) as a drug carrier for cancer chemotherapy. 10-Hydroxycamptothecin (HCPT) was encapsulated into HANP for in vitro and in vivo evaluations. HANP/HCPT demonstrated improved cytotoxicity* in vitro* on five cell lines as well as anticancer activity in vivo, which was monitored by ^18^F-PET imaging. No systemic toxic effects were observed. This work highlighted the potency of utilizing HANP as drug carrier for targeted tumor chemotherapy.

J. Zhang et al. review recent clinical applications of contrast-enhanced perfusion weighted (PW) MRI techniques in glioma theranostics. Due to complex and heterogeneous vasculatures of gliomas, the prognosis of gliomas remains poor although diagnostic techniques advance in recent years. Based on the current clinical evidence, PW-MRI is improving glioma prognosis, treatment, and response assessment although there are some clinical barriers and unsolved issues.

M. Iori et al. estimate manual, semiautomated, and fully automated synthesis for ^90^Y- and ^177^Lu-labelled radiopharmaceuticals in prostate cancer, one of the most prevalent cancers among men. The fully automated synthesis guaranteed reliable and reproducible preparations of pharmaceutical grade therapeutic radiopharmaceuticals although the potential concerns about radiation exposure to the operators still remain.

In summary, the six papers for this special issue containing 2 review articles and 4 original research reports, which were selected after peer-review by the guest editors Fu Wang, Lei Zhu, Kai Wang, and Dongkyoo Park, illustrate the broad scope of achievements and contributions of theranostics in the field of cancer research although all aspects of theranostics cannot be covered in one special issue.

